# Octahedral Dynamics and Local Symmetry in Hybrid Perovskite FAPbI_3_ under Thermal Excitation

**DOI:** 10.1021/acsomega.5c09719

**Published:** 2026-06-17

**Authors:** Himanshu Joshi, Kailash Chandra Bhamu, Amit Shankar, Rana Biswas, Mateusz Wlazło

**Affiliations:** † Department of Physics, SRM University Sikkim, Tadong, Gangtok 737102, Sikkim, India; ‡ Department of Physics, Mody University of Science and Technology, Lakshmangarh 332311, Rajasthan, India; § Condensed Matter Theory Research Lab, Department of Physics, Kurseong College, Darjeeling 734203, India; ∥ Electrical & Computer Engineering Department, 1177 Iowa State University, Ames, Iowa 50011, United States; ⊥ Chemical and Biological Systems Simulation Lab, Centre of New Technologies, University of Warsaw, Banacha 2C, 02-097 Warsaw, Poland

## Abstract

Density functional theory (DFT) and ab initio molecular dynamics (AIMD) simulations have been employed to investigate the evolution of local motifs within the tetragonal phase of FAPbI_3_ under thermal excitation. Our results reveal a distinct broadening in the distribution of PbI_6_ octahedral volumes with increasing temperature, indicating a gradual breakdown of symmetry and emergence of diverse local environments. These octahedral volume distortions are primarily driven by the dynamic behavior of the FA cation, leading to softening of PbI_6_ octahedra, evident from the calculated mean octahedral volume and Pb–I–Pb bond angles. The examination of the electronic structure confirmed that this dynamic structural phenomenon is directly responsible for the change in fundamental band gap value, highlighting the role of PbI_6_ octahedra in modifying and modulating the electronic properties in FAPbI_3_. The results demonstrate the microscopic origin of thermally induced dynamical behavior to the macroscopic electronic properties and underscore the pivotal role of local motifs in hybrid perovskites.

## Graphical abstract

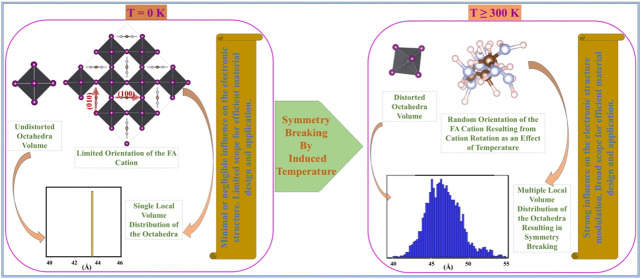

## Introduction

Organic–inorganic hybrid halide perovskite of the general formula APbX_3_ (A = CH_3_NH_3_ or CH­(NH_2_)_2_ and X = halide atom) has recently drawn widespread attention due to its superior solar power conversion efficiency.^1–3^ These perovskites outperform silicon and other conventional photovoltaic materials,^4,5^ making them particularly desirable for high-performance photovoltaic applications. Significant advancements have demonstrated that substituting methylammonium (MA) = CH_3_NH_3_ by a large formamidinium (FA) = CH­(NH_2_)_2_ cation yields remarkable power conversion efficiencies above 22%.^6^ This improvement results because FA increases the effective cation radius, leading to a reduction in the optical band gaps and thereby enhancing light absorption.^7–9^ In addition to this electronic influence, the FA cation plays a structural role by templating a network of corner-sharing PbX_6_ octahedra. Thus, the orientation and dynamic behavior of this molecular cation critically affect both stability and efficiency. Moreover, hybrid halide perovskites like FAPbI_3_ are known to exhibit complex phase transitions, which is highly sensitive to temperature and pressure.^10^ At ambient temperature, the compound stabilizes in a pseudocubic or α-phase,^11^ which transitions to tetragonal and subsequently to orthorhombic phase as temperature decreases.^9,12^ These lower temperature phases have a large band gap,^13^ making them poor solar absorbers and are therefore less explored in photovoltaic studies. However, the precise nature of phase transition and magnetic ordering in the low-temperature phases of FAPbI_3_ remains debatable.^14–17^ Experimental evidence suggests that the orientation of the molecular cation drives phase transition in the hybrid halide perovskites,^15^ although a detailed theoretical understanding of the organic molecule orientation dynamics in these materials are still lacking. Interestingly, some studies have addressed the inconsistencies between theoretical predictions and experimental findings by employing a symmetry-broken polymorphous network approach,^18,19^ particularly in band gap estimations and mixing enthalpies of cubic phase as well as other phases. Such networks, obtained by removing the standard restriction to a minimal unit cell size, allow for a variety of octahedral tilts and distortions, with significant lowering of calculated total energies.^20–22^ Although key aspects of polymorphous models and cation dynamics for certain quantum materials have been established, particularly in oxide perovskites,^18–20,22^ the influence of temperature on local structural motifs and their effect on the electronic properties of hybrid halide perovskite is not well understood and is a critical area for further study.^21^ Motivated by this, we investigated the role of temperature in driving octahedral distortions within the tetragonal phase of FAPbI_3_. A key factor is the complexity in the behavior of the A-site cation. Unlike in purely inorganic halide perovskites, where A-site is occupied by a spherical inorganic ion, the presence of an organic molecule introduces additional degrees of freedom. The FA cation, in particular, undergoes dynamic motions including dihedral rotations and high-frequency vibrations,^23^ which considerably influence both the local structural symmetry and properties of these materials.

In this work, we investigate the dynamical distortion in PbI_6_ octahedral volumes and the Pb–I–Pb bond angles, induced by FA molecular cation as a function of temperature. The electronic band structures were calculated at various temperatures to correlate the effect of PbI_6_ octahedral distortion with the change in electronic properties. To obtain a reasonable electronic structure of the large supercells used in our calculations, the band unfolding technique^24–27^ was implemented, which maps the electronic states of the supercell Brillouin zone onto that of the primitive cell. The 0 K ideal-symmetry structure exhibits lower band gap values than the temperature-induced symmetry-broken structures. We show that thermal disorder first breaks the symmetry, which increases the gaps, before the effect of octahedral distortion and expansions takes over. The FA cation orientation was examined up to a temperature range of 500 K, revealing that the cation rotates more freely with increasing temperature, providing direct evidence of the observed broader octahedral distortions resulting from breaking of the local symmetry. Ab initio molecular dynamics (AIMD) further demonstrates that the local motifs evolve continuously with temperature, leading to the emergence of a polymorphous network of octahedral tilts and distortions. The temperature-driven evolution of local structural motifs and their influence on the electronic properties of hybrid halide perovskite materials remains an area that has not been thoroughly investigated. These insights linking the microscopic origins of dynamic behavior to the macroscopic electronic properties in FA-based perovskites are crucial for the rational design of more efficient materials.

## Computational Methods

All calculations are based on the first-principles method and were performed employing the Vienna Ab Initio Simulation Package (VASP),^28,29^ incorporating projector augmented-wave (PAW) pseudopotentials^30^ and employing the SCAN^31^ exchange–correlation functional. The SCAN functional has shown improved accuracy in capturing structural and electronic properties especially in systems involving symmetry breaking,^20^ when compared to more conventional functionals such as PBE^32^ or even DFT + U^33^ approaches. The energy cutoff values were fixed at 500 eV for structural relaxation and increased to 550 eV for volume optimization and molecular dynamics (MD) simulations. Initial structural optimization was accomplished using a k-point density of approximately 1000 points per reciprocal atom, which was refined to 10,000 for the final geometry optimization. For AIMD simulations, calculations were carried out using only the Γ-point. Corresponding to the lattice parameters *a* = *b* = 9.08 and *c* = 12.68 Å, a 2 × 2 × 2 supercell containing 384 atoms was constructed and fully relaxed until all atomic forces fell below 0.01 eV/Å. To explore the structural effect with temperature, AIMD simulations were carried out under constant number of atoms (*N*), pressure (*P*), and temperature (*T*) conditions using the NPT ensemble. Temperature and pressure control were maintained using the Langevin thermostat in conjunction with the Parrinello–Rahman barostat.^34,35^ A time step of 1 fs was used throughout. The atomic degrees of freedom were subjected to a friction coefficient of 3 ps^–1^, while the lattice degrees of freedom were governed by a coefficient of 10 ps^–1^. Postprocessing and visualization of structural and electronic data were performed using the Python Materials Genomics (pymatgen)^36^ toolkit and the VESTA^37^ software suite. The computational approach adopted aligns with well-established methodology^20–22^ for simulating thermally driven symmetry breaking in complex systems.

## Results and Discussion

The tetragonal phase of FAPbI_3_ features corner-sharing octahedra, where Pb atoms occupy the B-site and are coordinated by six iodine neighbor atoms. These octahedra are a defining feature of the perovskite structure. We consider the conventional 4 f.u./cell minimal tetragonal structure^19,38^ and relax the atomic positions maintaining the symmetry. As previously discussed, to capture the full range of the possible octahedral distortions, including Glazer-type rotational modes like a^0^a^0^b^–^, structural relaxation on a 2 × 2 × 2 supercell containing 32 f.u./cell was considered, which is sufficient for effective inclusion of long-range interactions.^18,39^ The optimized tetragonal 32 f.u./cell structure showed limited FA molecular orientation only along the [100] or the [101] lattice direction and modest PbI_6_ tilting (Figure 1a), implying that the symmetry is not broken. We use this structural configuration to enable us monitor the progressive weakening of structural disproportionation, offering insight into the underlying mechanisms of structural disproportionation and shedding light on how temperature influences the arrangement of local motifs. With thermal excitation introduced by AIMD, the displacement in the Pb atomic sites induces tilting of the PbI_6_ octahedra, while the rotation of the FA molecule within its cage causes disproportionation among the octahedra (Figure 1b). Such symmetry breaking introduced by temperature severely affects the band gap of the material. It also causes the loss of structural bond, which changes the local environment in the crystal symmetry, leading to substantial energy lowering, thus stabilizing the structure.

**Figure 1:**
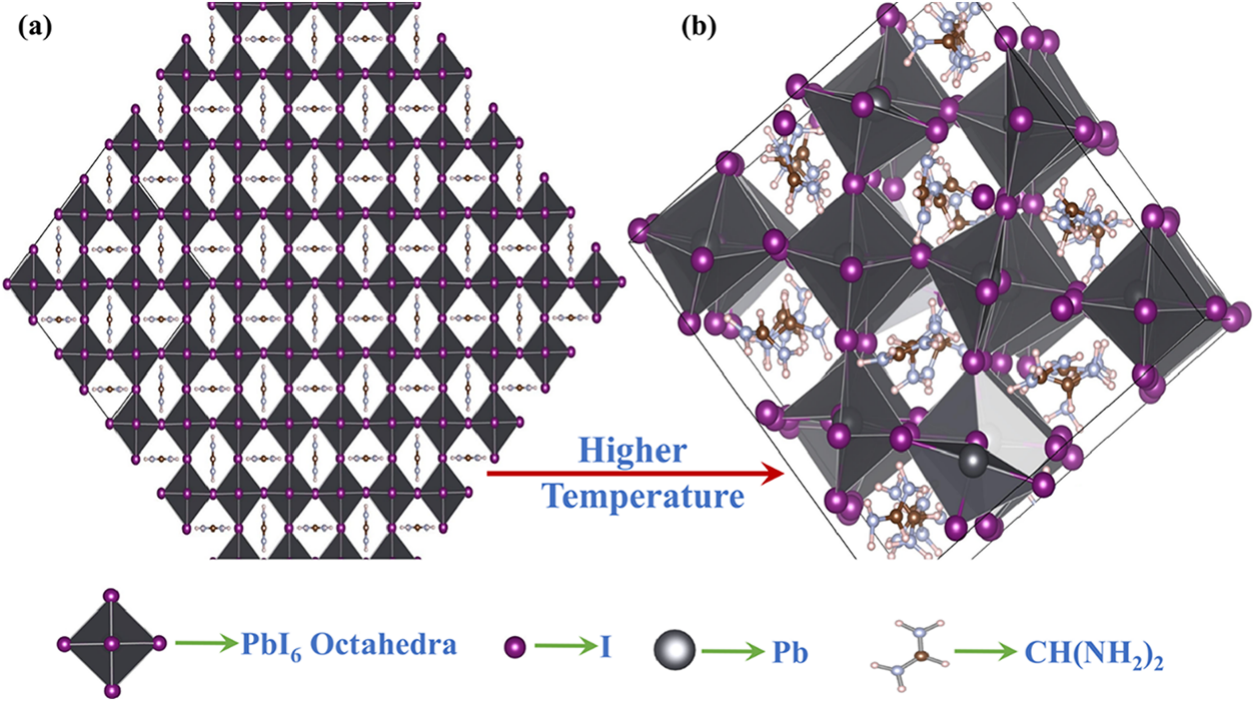
Effect of temperature on the FA molecular orientation and octahedral disproportionation. (a) Optimized tetragonal symmetry structure at 0 K with no octahedral tilting and minimal FA orientation. In 0 K, the cuboctahedra cages are extended along either the [100] or [010] orientation. (b) Snapshot of the structural evolution with temperature at 500 K. The crystal structure shows enhanced FA molecular rotation and octahedral tilting with the cuboctahedral cages at random orientation.

To investigate how temperature influences the arrangement of local structural motifs, ab initio molecular dynamics (AIMD) simulations were carried out within the NPT ensemble. Although related works have been conducted across various material systems,^40–42^ essential simulation parameters are often not clearly specified, particularly local motif distributions are based on very short AIMD runs, sometimes only a few picoseconds. We focus primarily on structural motifs, which can be effectively examined through experimental methods such as synchrotron X-ray diffraction (s-XRD)^43^ or pair distribution function analysis via local probes.^44^ Some studies report the ferromagnetic nature of low-temperature phases in FAPbI_3_ ^16,45^ and to investigate such behavior, spin motif distributions have to be considered. However, such investigations require more sophisticated theoretical treatment to capture the behavior of multiple magnetic sublattices. Therefore, spin motif distributions are not considered in the present discussion.

In AIMD simulations, a finite equilibration time is necessary to ensure the system reaches thermodynamic equilibrium.^46^ Conventionally, equilibrium is assessed by tracking the evolution of quantities, such as lattice constants, over time. Such plots of parameter evolution are crucial in understanding the physical state of the system with increasing temperature. As shown in Figure 2, minimal fluctuations in the cell parameter even at elevated temperatures, indicate the absence of lattice instability and confirm that the system does not attain an unphysical crystal structure due to lattice expansion as temperature rises.^20^ This further ensures that the broadening of octahedral volumes and bond angle distributions observed at higher temperatures arises from internal dynamic distortions rather than changes in global lattice parameters.

**Figure 2:**
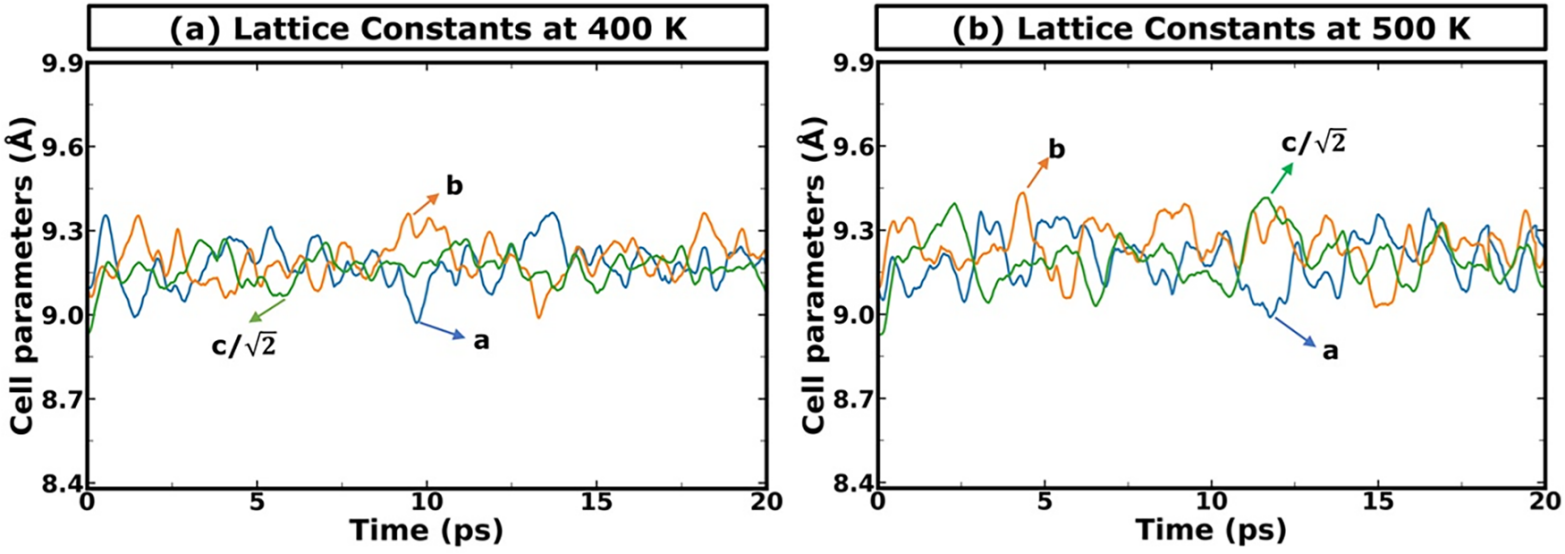
Ab initio molecular dynamics simulation of time evolution of lattice constant variations at higher ranges of temperature. The lattice constants evolution is calibrated up to 25 ps time, with the initial 5 ps reserved for system equilibrium and is hence omitted in the figure. To better visualize the lattice constants on the same numerical scale, the values of *a*, *b*, and *c*/√2 are plotted.

In the optimized 0 K structure, the system exhibits a uniform local environment with negligible variation in the local structural motifs, as evident in Figure 3. At this point, the PbI_6_ octahedra maintain a consistent volume, indicating a single, well-defined contribution to the structural framework. However, as the system is thermally excited, we begin to observe a noticeable distribution of these local motifs, quantified here through the calculated volumes of the PbI_6_ octahedra. At elevated temperatures, the previously singular peak in the PbI_6_ volume distribution broadens and shifts, suggesting that thermal fluctuations promote local distortions within the structure. This shift gradually evolves into a broad, Gaussian-like profile, marking the emergence of diverse local environments, as evident from the mean and standard deviation values of the octahedra volume obtained at different temperatures. Mean value of the octahedra volume distribution broadens with temperature, indicating softening of the octahedra due to tilting and stretching. For instance, the distribution observed at 500 K is significantly broader than that at 400 K, which, in turn, is broader than that at 300 K, clearly revealing a temperature-dependent expansion of local motif diversity. At 0 K, the observation of a single local environment likely stems from the fact that the initial structure was not symmetry broken. However, if symmetry breaking were induced at 0 K, implemented through a small perturbation to slightly shift atoms from their equilibrium lattice sites (polymorphous structure), the structure reveals at least a dual local environment, attributable to distortions in the PbI_6_ octahedra arising from the varied orientations of the FA cations confined within their cages.^47^ The undistorted octahedra volume calculated at 0 K was 43.57 Å^3^, which increases to a mean value of 46.58 Å^3^ at 500 K. This is the structural fingerprint of increased dynamical disorder that represents local symmetry breaking. To the best of our knowledge, no direct theoretical or experimental comparison on PbI_6_ octahedra volumes was obtainable from the literature; herein, we present the first comprehensive analysis.

**Figure 3:**
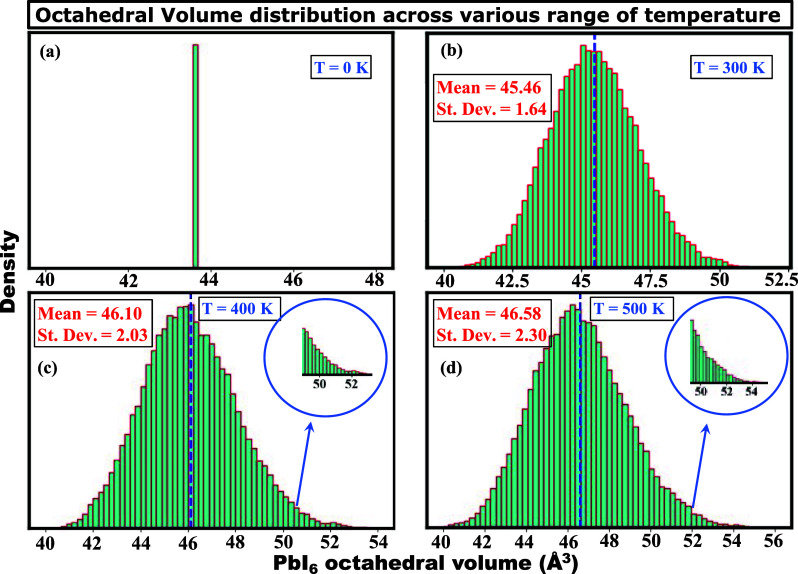
PbI_6_ octahedral volume distributions at various temperatures, sampled over 20 ps following an initial 5 ps equilibration period. (a) Single local environment denoted by a single volume of the PbI_6_ octahedra. (b–d) Broadening of the octahedral volume with temperature. The broadening indicates change in the local crystal environment with temperature. The figure insets in (c) and (d) highlight the enhanced volume distribution induced by temperature.

Understanding the nature of these distributions, especially how they evolve with temperature, can offer key insights into the mechanisms driving disproportionation in hybrid perovskites. The broadened distribution at higher temperatures suggests a dynamic restructuring of local environments, potentially underpinning the material’s phase evolution.^20,47^ While AIMD simulations offer a useful approximation of these behaviors, the absolute volume values should be interpreted cautiously; what holds greater significance is the pattern and extent of the distribution itself. Furthermore, capturing such subtle symmetry breaking features experimentally would require advanced local probes, as conventional X-ray diffraction techniques may not be sensitive enough to detect them. This highlights the importance of complementary methods such as pair distribution function (PDF) analysis, which can more effectively reveal hidden local structural variations and provide a more comprehensive understanding of the material’s thermally driven transformations.

To understand the evolution of band structure with this dynamical distortion, unfolded band structure plotted in the first Brillouin zone across various ranges of temperature is shown in Figure 4. The effective elemental contribution as well as the direct nature of the gap remains unchanged with the introduction of temperature. However, a nonmonotonic evolution of band gap is observed, *E*_g_ (0 K) < *E*_g_ (500 K) < *E*_g_ (400 K) < *E*_g_ (300 K), highlighting the profound impact of octahedral distortion on the electronic landscape. At 0 K, the high-symmetry structure yields well-defined unfolded electronic states with a direct gap of 1.53 eV along the Γ-symmetry point, resulting in clean bands and aligns with the available literature.^48^

**Figure 4:**
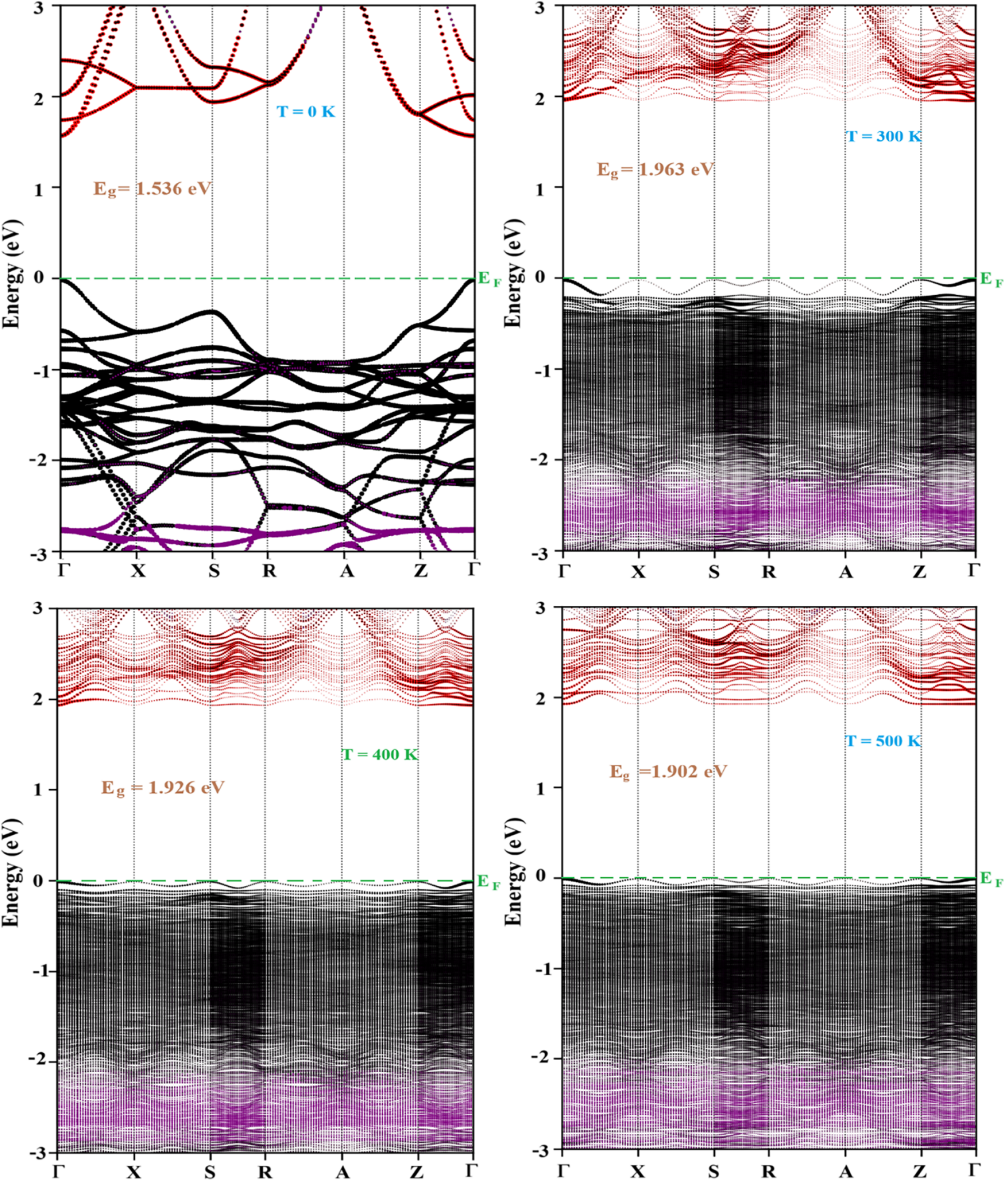
Unfolded effective band structure of 2 × 2 × 2 supercell containing 32 f.u./cell at 0 K, 300 K, 400 K, and 500 K. The color variation in the unfolded band structure reflects the elemental contributions, where red indicates Pb-derived states and black represents I-derived states at the band edges.

As the temperature increases, a significant broadening of the band edges is observed. The highly smeared band edges provide a direct signature of the loss in symmetry induced by octahedral volume distortion and anharmonic FA cation motion. The FA molecule reorients freely, broadening the volume distribution (Figure 3) as temperature increases, resulting in a significant narrowing of the band gap with increasing dynamic disorder. This trend therefore reflects the competition between dynamic symmetry breaking, which transiently widens the gap, and disorder-induced distortions, which counteract this effect by reducing the gap at higher temperatures. Also, a significant decrease in the Pb–I–Pb bond angle is observed as a result of dynamic disorder, as shown in Figure 5. These angles define the volume of the cuboctahedra cavity, where the ideal value of 180° corresponds to the most ordered cubic symmetry characterized by collinear bonds in Pb–I–Pb.^49^ In the absence of dynamical disorder, higher values of the bond angle are associated with the lower values in the band gap,^49^ as observed from our 0 K results. However, as temperature increases, the Pb–I–Pb bond angle distribution becomes broader, shifting toward lower values due to enhanced rotational freedom of the FA molecule. This is reflected by the systematic reduction in both the mean and median bond angles with increasing temperature. The broadening indicates the presence of multiple instantaneous PbI_6_ octahedral tilt configurations. The additional rotational freedom induces further distortion to the PbI_6_ octahedra, which in turn enhances FA molecular tilt angles and generates dynamic disorder, characterized by broadening of the octahedral volumes, as evident from Figure 3.

**Figure 5:**
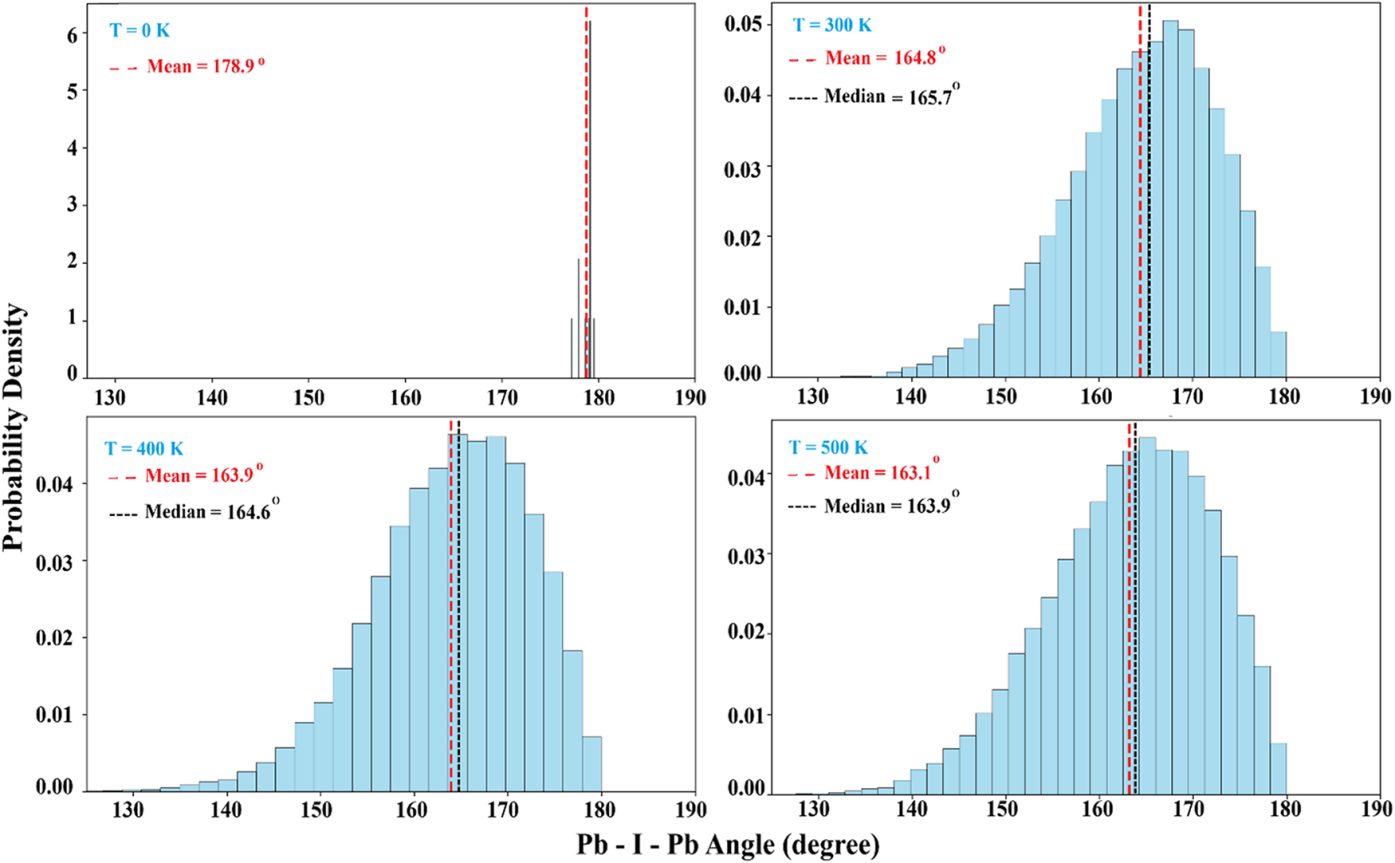
Evolution of Pb–I–Pb bond angle with temperature sampled over 20 ps followed by an initial 5 ps equilibration period.

The band gap behavior between 300 and 500 K temperature is attributed to the local structure disproportionation-driven dynamic disorder. Interestingly, the band gap at 300 K is larger in comparison to 0 K. This is primarily due to enhanced symmetry breaking induced at 300 K, a behavior consistent with earlier reports.^50^ The band gap widens because the PbI_6_ octahedral volume evolves from a sharp narrow peak at 0 K to a broadened, diverse local motif environment distribution at 300 K (Figure 3). The significant nonrigid breathing-mode distortion breaks the local symmetry lifting band-edge degeneracies that widens the band gap. As the temperature increases (400–500 K), thermal expansion takes over along with deformations in the PbI_6_ octahedra (octahedral softening), caused by increasing temperature. This reduces the Pb–I electronic coupling, which leads to lowering the conduction band minimum, thereby narrowing the band gaps. Thus, the observed band gap characteristics arise from two competing mechanisms: dominance of symmetry breaking at 300 K and the increasing influence of disorder-driven octahedral softening at higher temperature, which counteracts the induced gap widening.

## Conclusion

In this study, density functional theory combined with ab initio molecular dynamics simulation (AIMD) was employed to study the distribution of structural motifs in the tetragonal phase of FAPbI_3_. The study shows the microscopic origin of structural transformation that results in transforming and modulating the energy landscape of electronic properties. Our results show that symmetry breaking induced by thermal excitation broadens the PbI_6_ octahedral volume and the Pb–I–Pb bond angle due to enhanced FA cation orientation. These octahedral distortions give rise to a nonmonotonic temperature-induced band gap characteristic, with spectral blue shift at 300 K, associated with a diverse local environment due to symmetry breaking. The electronic structure investigation reveals that this dynamic structural phenomenon is directly responsible for modifying and modulating the electronic properties in FAPbI_3_. Our findings highlight the role of local motifs in governing the electronic properties of hybrid perovskites, providing valuable insights for the rational design of more efficient materials.
